# Reproducibility of Bacterial Cellulose Nanofibers Over Sub-Cultured Generations for the Development of Novel Textiles

**DOI:** 10.3389/fbioe.2022.876822

**Published:** 2022-04-25

**Authors:** Jane Wood, Christopher van der Gast, Damian Rivett, Joanna Verran, James Redfern

**Affiliations:** ^1^ Manchester Fashion Institute, Faculty of Arts and Humanities, Manchester Metropolitan University, Manchester, United Kingdom; ^2^ Department of Life Sciences, Faculty of Science and Engineering, Manchester Metropolitan University, Manchester, United Kingdom; ^3^ Department of Natural Sciences, Faculty of Science and Engineering, Manchester Metropolitan University, Manchester, United Kingdom

**Keywords:** bacterial cellulose, *Komagataeibacter xylinus*, pellicle, textiles, sustainability

## Abstract

The textile industry is in crisis and under pressure to minimize the environmental impact on its practices. Bacterial cellulose (BC), a naturally occurring form of cellulose, displays properties superior to those of its cotton plant counterpart, such as enhanced purity, crystallinity, tensile strength, and water retention and is thus suitable for an array of textile applications. It is synthesized from a variety of microorganisms but is produced in most abundance by *Komagataeibacter xylinus*. *K. xylinus* is available as a type strain culture and exists in the microbial consortium commonly known as Kombucha. Whilst existing literature studies have described the effectiveness of both *K. xylinus* isolates and Kombucha in the production of BC, this study investigated the change in microbial communities across several generations of sub-culturing and the impact of these communities on BC yield. Using Kombucha and the single isolate strain *K. xylinus* as inocula in Hestrin and Schramm liquid growth media, BC pellicles were propagated. The resulting pellicles and residual liquid media were used to further inoculate fresh liquid media, and this process was repeated over three generations. For each generation, the thickness of the pellicles and their appearance under SEM were recorded. 16S rRNA sequencing was conducted on both pellicles and liquid media samples to assess changes in communities. The results indicated that the genus *Komagataeibacter* was the most abundant species in all samples. Cultures seeded with Kombucha yielded thicker cellulose pellicles than those seeded with *K. xylinus*, but all the pellicles had similar nanofibrillar structures, with a mix of liquid and pellicle inocula producing the best yield of BC after three generations of sub-culturing. Therefore, Kombucha starter cultures produce BC pellicles which are more reproducible across generations than those created from pure isolates of *K. xylinus* and could provide a reproducible sustainable model for generating textile materials.

## Introduction

The textile industry is a major contributor to greenhouse gas emissions, global water consumption, and contamination (Ellen MacArthur Foundatio, 2017). On its current trajectory alongside a heavy reliance on non-renewable resources, the industry will be responsible for 26% of the carbon budget associated with a 2°C global temperature increase by 2050. Under pressure to increase sustainability and minimize the environmental impact on its practices, the textile industry is seeking alternatives to meet global demands (Textiles 2030 project, 2021), with replacements for traditional fiber sources being one area of exploration.

One such traditional fiber cotton accounts for approximately one-third of fibers used worldwide, and the demand is expected to grow due to the versatility of its cellulosic base (Voora et al., 2020). This plant-based cellulosic fiber has a huge environmental impact; it requires a large volume of water to support its growth to maturity, alongside the use of environmentally harmful pesticides and chemicals to achieve high yield and good quality crops (Blackburn, 2009; Stone et al., 2020). Additionally, the cotton fiber structure contains contaminants (for example, non-cellulosic proteins, amino acids, and waxes) which have a negative impact on the end use and need to be removed *via* extensive cleaning processes (Sinclair, 2015).

In contrast to its plant counterpart, bacterial cellulose (BC) is a form of naturally occurring cellulose in a highly pure state (Lin et al., 2013). It possesses properties such as a highly crystalline structure, high tensile strength, and considerable water retention behavior (Dufresne, 2012). As such, this makes it suitable for medical applications (wound dressings, artificial skin, and tissue engineering) (Hoenich, 2006; Bodin et al., 2007; Bäckdahl et al., 2008; Gatenholm and Klemm, 2010; Huang et al., 2014; Picheth et al., 2017; Torres et al., 2019; Azimi et al., 2021), cosmetics and beauty care (Amnuaikit et al., 2011; Mohite and Patil, 2014), food and packaging (Chawla et al., 2009; Kuswandi et al., 2014; Shi et al., 2014; Ullah et al., 2016; Ashrafi et al., 2017), acoustics (Iguchi et al., 2000), paper (Lee, 2018; Torres et al., 2019), electrical energy storage and sensors (Hu et al., 2011; Jia et al., 2016), filtration, absorbents and adsorbents (Mohite and Patil, 2014; Stoica-Guzun et al., 2016; Zhu et al., 2016), and fabrics for apparel (Fernandes et al., 2019; García and Prieto, 2019; Lee, 2019).

As a biopolymer, BC is reportedly synthesized by a variety of microorganisms (Dufresne, 2012). Studies suggested that it is the *Komagataeibacter* genus (formerly known as *Gluconacetobacter* and *Acetobacter*) that produces BC in the greatest abundance (Yamanaka and Sugiyama, 2000; Jayabalan et al., 2014), while *Komagataeibacter xylinus is* widely accepted as the most productive.

In a liquid medium with a carbon source, *K*. *xylinus* develops a gelatinous biofilm, or pellicle, on the surface of the liquid, which is reported to consist of BC nanofibrils and extracellular material (Torres et al., 2019). In laboratory work, “standard” complex liquid media are often used to produce BC from a pure isolate of *K. xylinus*. The two most commonly used media consist of yeast extract, glucose and bactopeptone (known as Hestrin and Schramm media) Schramm and Hestrin (1954); (Ovcharenko, 2013) or sucrose, potassium phosphate, magnesium sulfate, and ammonium sulfate (known as Yamanaka media) Yamanaka and Sugiyama (2000). *K. xylinus* can be isolated from rotting fruit, as well as from bacterial and yeast communities such as Kombucha.

Kombucha, also known as Manchurian mushroom, Haipao, or tea fungus, has been used in the fermentation of drinks dating back to several thousand years (Jarrell et al., 2000; Teoh et al., 2004; Četojević-Simin et al., 2012) and is purported to have detoxifying and energizing properties when imbibed (Teoh et al., 2004; Marsh et al., 2014). The Kombucha “tea fungus”, or pellicle, can be considered a “core” consortium of bacteria and yeasts, with its exact composition determined by its geographic and climatic conditions of cultivation (Chakravorty et al., 2016). It is thought that the additional “local” bacteria and yeasts have some effect on the growth behavior of the overall community (Jayabalan et al., 2014) and that the microbial community composition can vary with fermentation time. It has been widely documented that the brewing of Kombucha tea for more than 3 days can lead to the “core” consortium producing a BC pellicle on the surface. Similar production is observed when Kombucha is used to inoculate standardized microbiological media such as Hestrin and Schramm (commonly known as H&S) or Yamanaka broth (Schramm and Hestrin 1954; Jarrell et al., 2000; Yamanaka and Sugiyama 2000; Chakravorty et al., 2016).

The impact of different growth liquids and different starter cultures has been demonstrated in the literature with respect to bacterial cellulose yield and changes in bacterial communities. Marsh et al. (2014) used Kombucha pellicles as inoculants from different geographical locations and analyzed the microbial communities present after 3 and 10 days of fermentation in black tea using metagenomic DNA extraction and high-throughput sequencing techniques and found that in all cases, *Komagataeibacter* was the dominant bacterial genus, with the highest diversity of microbial strains found in the cellulosic pellicles. Similar data were identified by Chakravorty et al. (2016) where Kombucha pellicles and liquids were assessed at 3-, 7-, 14-, and 21-day fermentation. *Komagataeibacter* was the dominant bacterial genus in both the liquid and pellicle at all time-points with microbial diversity declining throughout the study.

When examining microbial communities in Kombucha tea, Reva et al. (2015) also discovered diversity was found to be less in the pellicles and more in the liquid phase. Reva et al. (2015) postulated more work is required to evaluate the core species in Kombucha that are responsible for pellicle formation and that this could be used as a platform to assess potential end uses of the formed pellicles.

However, to date, there is little research to examine the changes in the bacterial communities in both the liquid and pellicle over several generations of sub-culturing, where previous studies have instead focused on the age of a single culture. If BC is going to provide the textile industry with a more sustainable model for generating materials, it is essential that the effect of sub-culturing over numerous generations is understood as reproducibility would be an essential requirement for any industrial use. Therefore, the aim of this study was to analyze the effect of multi-generational sub-cultures of BC pellicles and the liquid environment it grows in, with respect to physical properties and microbial community composition and changes.

## Materials and Methods

### Starter Cultures

This study used either *Gluconacetobacter xylinus* (ATCC 23767) (now named *Komagataeibacter xylinus*) as a single isolate strain or a Kombucha starter culture (KSC—www.happykombucha.co.uk) to propagate bacterial cellulose over three generations. *K*. *xylinus* was stored at −80°C in a cryopreservative solution [3.6% KH_2_PO_4_, 12.6% K_2_HPO_4_, 0.9% Na_3_C_6_H_5_O_7_, and 1.8% (NH_4_)_2_SO_4_]. When required, it was grown on H&S agar overnight at 30°C and was then used to inoculate a liquid medium for experiments detailed as follows. KSC, as provided by suppliers, comprises a solid surface (hereafter termed “pellicle”) and liquid phase. Storage of KSC was at room temperature in the absence of light in a sealed container, following manufacturer’s instructions.

### Growing Bacterial Communities Across Generations

**FIGURE 1:**
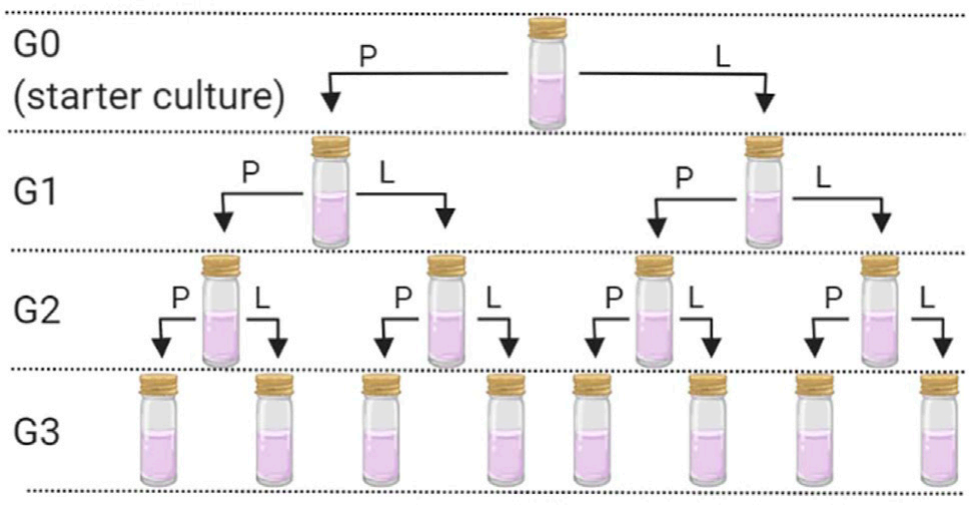
Generation overview and sampling scheme. P = use of the pellicle for the inoculum and L = use of the liquid phase for the inoculum. This process was followed for each of the two starter cultures. Following G0, each inoculation was carried out in duplicate.

Sterile “universal” tubes (25 ml) containing 10 ml of sterile H&S broth were inoculated with either 1 ml of *K. xylinus* or 1 g of KSC pellicles. The tubes were covered with loose-fitting lids and left at 30°C to incubate for 10 days—producing generation zero (G0). Following the 10-day incubation, pellicles had formed at the liquid–air interface in both starter cultures. Each pellicle was removed aseptically and divided using a sterile scalpel. Half of each pellicle was frozen at −80°C for DNA extraction and analysis (see below). The other half of the pellicle was further divided into two and used as the inoculum for generation 1 (in duplicate) 10 ml sterile H&S broth (G1P; Figure 1). Further duplicate G1 cultures were established using 10 ml of H&S broth that had been inoculated with 1 ml of the G0 liquid phase (G1L; Figure 1). This resulted in eight new starter cultures, inoculated from either the pellicle or liquid phase for both the community started by *G. xylinus* or the KSC. These cultures were incubated at 30°C for 30 days. After 30 days, the pellicle and broth were removed and sampled as mentioned earlier. The process was repeated to create generation 2 (G2) and generation 3 (G3), where each of the previous generation bacterial communities resulted in two new inoculations (one from the pellicle and one from the liquid phase) (Figure 1).

### Physical Quantification of Samples

At the end of each generation, before the pellicle was removed from the pot, the thickness was measured, using a ruler on the outside of the pot, and recorded. Pellicles were photographed both before and after removal from the pot, and visual appearance observations (color and opacity/transparency) were noted.

Samples of all pellicles were prepared for scanning electron microscopy (SEM) analysis by immersing each sample in 0.1 M glutaraldehyde overnight (to fix and preserve the cell structure), removing, and then dehydrating by passing through sequential ethanol baths (10 min per bath) of increasing concentrations (50%/70%/80%/90%/99.9%). The samples were kept in a desiccator until ready to be viewed on the SEM (Carl Zeiss Ltd., model Supra 40VP).

### Molecular Analysis of Microbial Communities in Samples

Nucleic acid extractions were carried out using the DNeasy PowerSoil kit (QIAGEN Ltd., Manchester), cleaned using the ZR-96 DNA Sequencing Clean-Up kit (Zymo Research, United States), and quantified using the Qubit dsDNA high sensitivity assay kit (Invitrogen, Paisley).

Amplicon sequencing of bacterial ribosomal rRNA genes (16S rRNA) was undertaken, as previously described (Thompson et al., 2017). Briefly, PCR reactions for initial amplifications consisted of 5 µl NEB Q5 reaction buffer, 0.25 µl NEB Q5 High-Fidelity DNA Polymerase, 0.5 µl NEB X nM dNTPs, 15 µl DNA template (c. X ngµl^−1^), and 0.5 µl X pM primers 515F (5′-GTGYCAGCMGCCGCGGTAA-3′) and 806_R (5′-GGACTACNVGGGTWTCTAAT-3′) or EUK1391_F (5′-GTA​CAC​ACC​GCC​CGT​C-3′) and 1510_R (5′-CCTTCYGCAGGTTCACCTAC -3′) for 16S and 18S rRNA genes, respectively, and made up to a final reaction volume of 25 µl with nuclease-free water. Cycling conditions comprised an initial denaturation at 94°C for 3 min, followed by 35 cycles of denaturation at 94°C for 45 s, annealing at 50°C for 60 s, and extension at 72°C for 90 s, with a final extension at 72°C for 10 min. Amplification was confirmed visually by 1.5% (w/v) Tris-acetate-EDTA (TAE)-agarose gel electrophoresis. A second-stage PCR was carried out to attach barcodes for Illumina sequencing. The constituents of the second-stage PCR reaction are as follows: 10 µl NEB Q5 reaction buffer, 0.5 µl NEB Q5 High-Fidelity DNA Polymerase, 1 µl NEB (X nM) dNTPs, 0.5 µl (X pM) forward primer, 0.5 µl (X pM) reverse primer, and 20 µl cleaned amplicon template, and they were made up to a final reaction volume of 50 µl with nuclease-free water. Cycling parameters comprised an initial denaturation at 98°C for 30 s, followed by 10 cycles of denaturation at 98°C for 10 s, annealing at 62°C for 20 s, and extension at 72°C for 30 s, with a final extension at 72°C for 2 min. Amplification and attachment were confirmed by 1.5% (w/v) TAE-agarose gel electrophoresis.

Following second-stage PCR, all amplified DNA strands were normalized using the SequalPrep Normalization kit (Fischer Scientific, Loughborough) and pooled into libraries for sequencing on a MiSeq Illumina platform with a MiSeq Reagent Kit v2 (300-cycle) flow cell.

### Data Analysis

Amplicon sequence variants (ASVs) were extracted from the raw sequence data using the dada2 pipeline (Callahan et al., 2016) using the default parameters. Taxa were assigned using the SILVA database and sequencing information files deposited in the NCBI BioProject database (BioProject PRJNA787576; accession numbers SAMN23827663–SAMN23827813). Statistical analysis is described in the results section, with α set at 0.05. All assumptions for parametric statistics were assessed visually prior to analysis. Multivariate ordination plots were calculated using the Bray–Curtis dissimilarity measure and non-metric multidimensional scaling (NMDS). Analysis of similarity (ANOSIM) was used to compare compositional differences in communities between two groups, with permutational ANOVAs (PERMANOVAs) used to analyze differences between communities as a factor of multiple factors using Bray–Curtis dissimilarity measures in the vegan package (v.3.5-0) (Oksanen et al., 2016).

Comparisons of community shifts were achieved using multivariate ANOVAs (MANOVAs). Univariate analyses were performed using repeated measures ANOVA (linear mixed-effects modeling fit by REML) in the lme4 (Bates et al., 2015) package, with significance assigned from the lmerTest (Kuznetsova et al., 2017) package, where generation was both a random effect and a fixed effect along with the original seed culture and phase measured as fixed effects.

## Results and Discussion

### Physical Characterization

**FIGURE 2:**
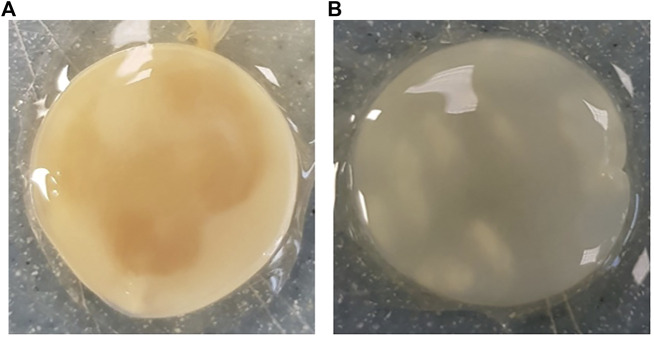
Examples of pellicles (approx. 2 cm across) produced in generation 2 from KSC **(A)** and the *K. xylinus* isolate **(B)**.

Across all generations, the Kombucha (KSC) pellicles had a consistent pale brown gel-like appearance, as noted from previous studies (Jarrell et al., 2000; Domskiene et al., 2019). However, previous work has not highlighted the visual variations our study found in the *K. xylinus* pellicles. In contrast to the consistent KSC pellicles, white, opaque spots were observed within the *K. xylinus* pellicle structure, as indicated in Figure 2.

In contrast to the KSC pellicles, *K. xylinus* pellicles, from both inoculum types, were initially (generation 1) translucent with a gelatinous appearance and were difficult to remove from the vessel due to their gel-like adherent consistency. In both second and third generations, however, the pellicles developed regions of opaqueness and were more robust.

Pellicle thickness increased as the generations progressed (Table 1), from 1 mm in all samples observed in generation 1 to between 2.4 mm *K. xylinus* pellicles (KXP) and 3.9 mm Kombucha pellicles (KP) in generation 3, despite all generations having the same incubation time. This trend was observed regardless of the starter culture. However, the inoculum type had an effect on the pellicle yield in generation 3; the highest yields by the thickness of pellicle were achieved when a liquid phase inoculum had been used at some stage of the process, with a lower G3 yield when only pellicle phase inocula had been used throughout the preceding generations (Table 1). Dima et al. (2017) and Blanco Parte et al. (2020) noted that the bacterial communities responsible for BC production are more mobile in the liquid phase and have greater access to nutrients, thereby improving their BC production potential and suggesting a reason for the observed differences in pellicle thickness. This leads to the hypothesis that liquid inocula achieve higher yields of pellicles, with Kombucha-derived liquid inocula producing higher yields than pure strain *K. xylinus.*

**TABLE 1:** Observed mean thickness of pellicles.

Starter culture	Generation 1			Generation 2			Generation 3		
KSC	KP	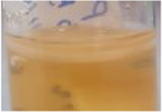	0.5 mm (0)	KP	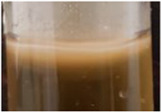	2.0 mm (0)	KP	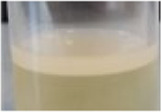	2.5 mm (1.6)
							KL	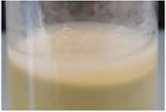	3.0 mm (1.8)
				KL	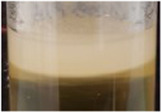	4.0 mm (0)	KP	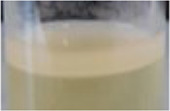	3.9 mm (1.3)
							KL	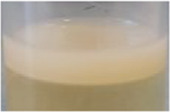	4.0 mm (1.6)
	KL	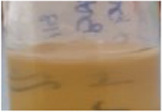	0.5 mm (0)	KP	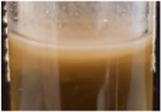	2.0 mm (0)	KP	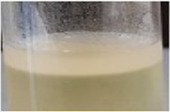	2.6 mm (0.4)
							KL	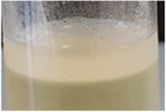	3.9 mm (0.9)
				KL	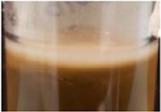	2.0 mm (0)	KP	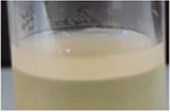	3.8 mm (1.8)
							KL	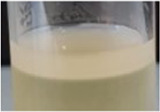	4.1 mm (1.4)
KXSC	KXP	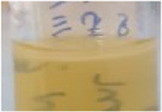	0.5 mm (0)	KXP	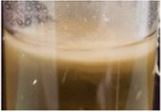	3.0 mm (1.0)	KXP	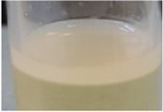	2.6 mm (1.5)
							KXL	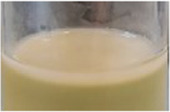	1.8 mm (0.4)
				KXL	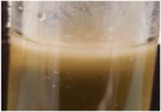	2.0 mm (0)	KXP	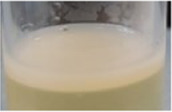	2.8 mm (1.0)
							KXL	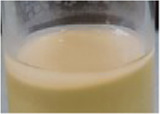	2.3 mm (0.4)
	KXL	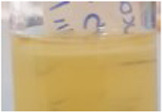	0.5 mm (0)	KXP	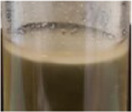	2.0 mm (1.0)	KXP	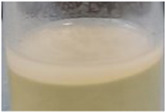	2.8 mm (0.8)
							KXL	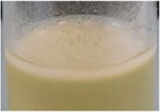	2.3 mm (0.4)
				KXL	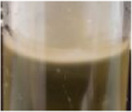	2.0 mm (0)	KXP	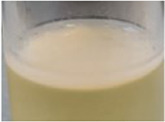	2.4 mm (0.4)
							KXL	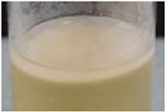	2.5 mm (0.5)

KSC, Kombucha starter culture; KXSC, *K. xylinus* starter culture; KP, Kombucha pellicle; KB, Kombucha liquid; KXP, *K. xylinus* pellicle; KXL, *K. xylinus* liquid. Generation 1, *n* = 1; Generation 2, *n* = 2; Generation 3, *n* = 8.

Additionally, previous studies of the fermentation of the Kombucha tea state that the yeast species present in the starter culture are instrumental in making carbon sources freely available (by breaking down complex sugars into more simple molecules) and in increasing the acidity of the broth as a by-product of the metabolic process (Teoh et al., 2004; Marsh et al., 2014). Both are favored conditions for BC-producing bacteria. Thus, the presence of yeast in the Kombucha created an advantage over the single isolate in the development of BC pellicles, particularly in broths such as H&S used in our study where there is no adjustment of pH. Furthermore, Jefferson, (2004) suggested there may be an ability to “store” carbon sources as extracellular polymeric substances (EPS) within the bacterial cellulose fibrillar matrix, thus creating a supply of nutrients which can be utilized when the source in the growth broth is depleted. This could explain the more rapid and consistent development of pellicles from Kombucha starter cultures. Here, a sequestered carbon supply is present when Kombucha is cultured compared to a single isolate where the nutrient source declines.

### Characterization by Scanning Electron Microscopy

**FIGURE 3:**
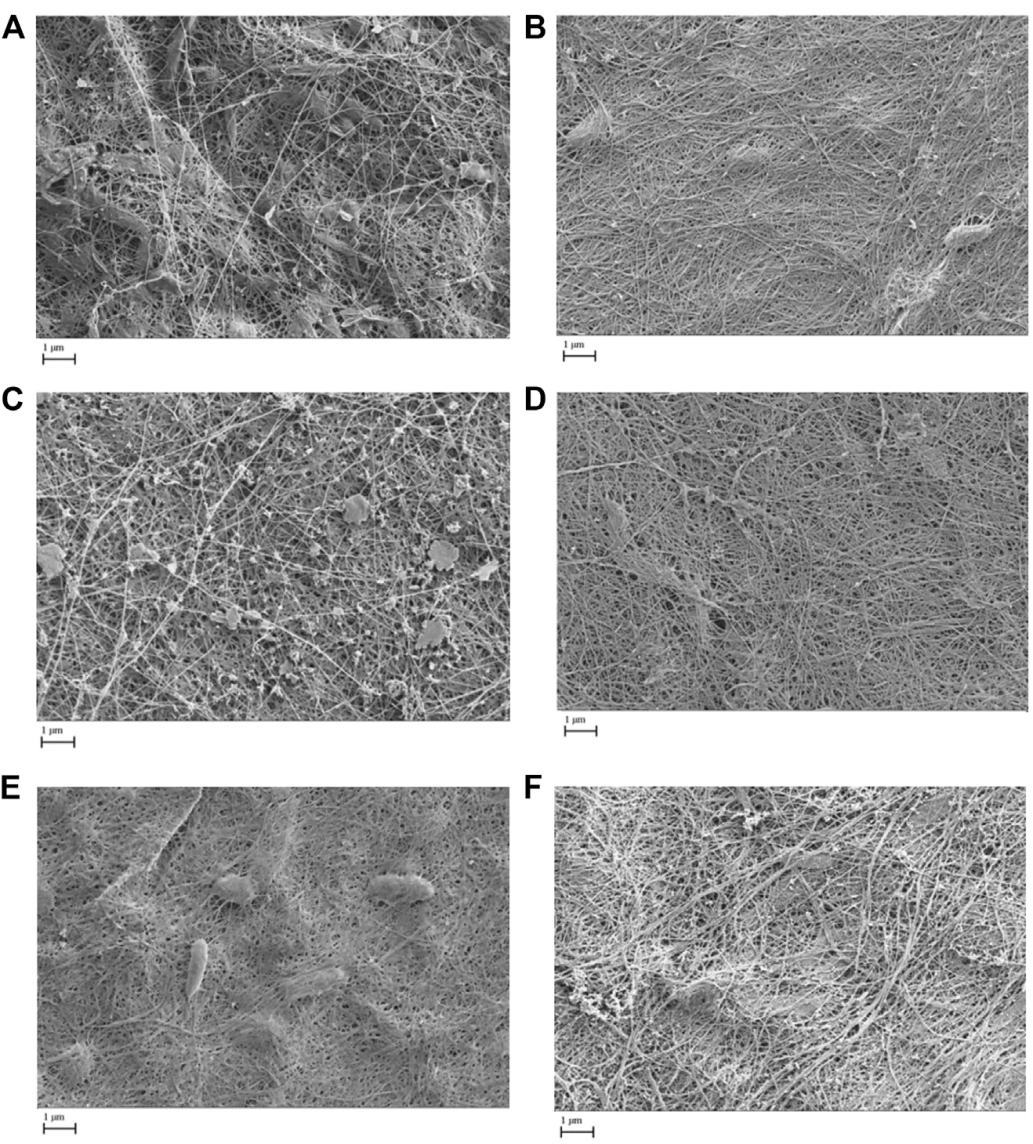
Scanning electron microscope images (x 20k) of generation 3 pellicles showing microfibrillar mat formation from various inoculants (**A** = Kombucha pellicle, **B** = *K. xylinus* pellicle, **C** = Kombucha broth, **D** = *K. xylinus* broth, **E** = Kombucha pellicle and broth, **F** = *K. xylinus* pellicle and broth).

All pellicles were examined by scanning electron microscopy. Figure 3 shows the comparisons of generation 3 pellicles as examples, illustrating similarities in the microfibrillar structure. However, SEM revealed pellicles developed from *K. xylinus* tended to develop more consistent and smoother microfibrillar mats. The pellicles developed from the Kombucha culture, whilst exhibiting a microfibrillar structure, also displayed a degree of contamination, which were suggested to be extracellular polymeric substances and a documented part of the Kombucha microbial consortium (Jefferson, 2004). Additionally, all pellicles showed similar size and distribution of nanofibrils, ranging from a (all broth inoculant) mean diameter of 81 (+/− 7.8) nm in *K. xylinus* to (mixed inoculant) a mean diameter of 92.5 (+/− 6.6) nm in Kombucha. The measured nanofibril size is in accordance with the findings of previous studies (Castro et al., 2011; Kumbhar et al., 2015; Chen et al., 2017). Furthermore, there were no other observed differences between the pellicle structures of those formed from liquid, solid, or mixed inocula.

### Community Abundance

The analysis of the 16S rRNA gene sequence data indicated that *Komagataeibacter* was the dominant genus in all samples, agreeing with findings of the previous work (Jarrell et al., 2000; Marsh et al., 2014; Gaggìa et al., 2019). The results indicate that pellicles inoculated with Kombucha (98.31% range: 44.09–99.99%) demonstrated a consistent (Wilcoxon rank sum test: W = 2,986 and *p* = 0.614) relative abundance of *Komagataeibacter* compared to those from a *K. xylinus* starter (97.70% range: 49.12–100%) (Tables 2, 3).

**TABLE 2:** Median ASV relative abundancies of *Komagataeibacter* in pellicles across generations.

Genus	G0		G1		G2		G3	
	GX^a^	K^a^	GX^a^	K^a^	GX^b^	K^b^	GX^c^	K^c^
*Komagataeibacter*	99.31 (99.22–99.39)	92.93 (91–72–94.13)	97.54 (95.74–99.17)	98.81 (98.07–99.56)	99.69 (93.81–99.96)	99.77 (70.40–99.99)	94.81 (49.13–100.00)	99.57 (44.09–99.97)

a *n* = 2.

b *n* = 16.

c *n* = 32.

**TABLE 3:** Median ASV relative abundancies of *Komagataeibacter* in liquids across generations.

N	G0		G1		G2	
	GX^a^	K^a^	GX^b^	K^b^	GX^c^	K^c^
*Komagataeibacter*	100 (67.51–100)	79.53 (68.72–98.30)	68.07 (61.11–99.17)	93.87 (91.30–94.27)	94.59 (64.78–100)	85.60 (50.13–96.00)

a *n* = 2.

b *n* = 4.

c *n* = 16.

**FIGURE 4:**
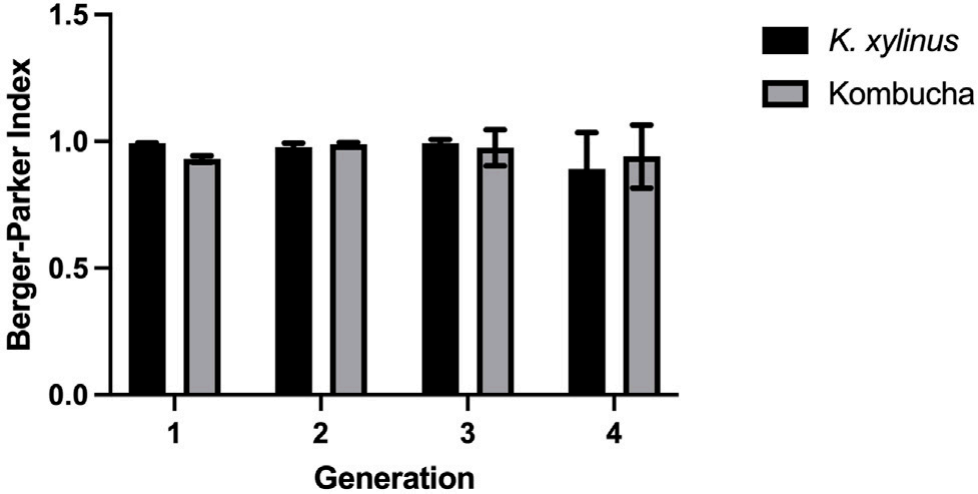
Relative abundance of dominant species (*Komagataeibacter*) across generations using the Berger–Parker index.

Additionally, the proportional abundance of *Komagataeibacter* in the pellicles was analyzed using the Berger–Parker index to establish the numerical importance of this as the most abundant species. Figure 4 illustrates the relative abundance of *Komagataeibacter* across generations of pellicles inoculated with either *K. xylinus* or Kombucha. Whilst *Komagataeibacter* is abundant in early generations of pellicles inoculated with *K. xylinus,* the trend of abundance slightly decreases over subsequent generations. Conversely, in pellicles inoculated with Kombucha, the trend of abundance of *K. xylinus* increases in later generations.

### Community Composition

**FIGURE 5:**
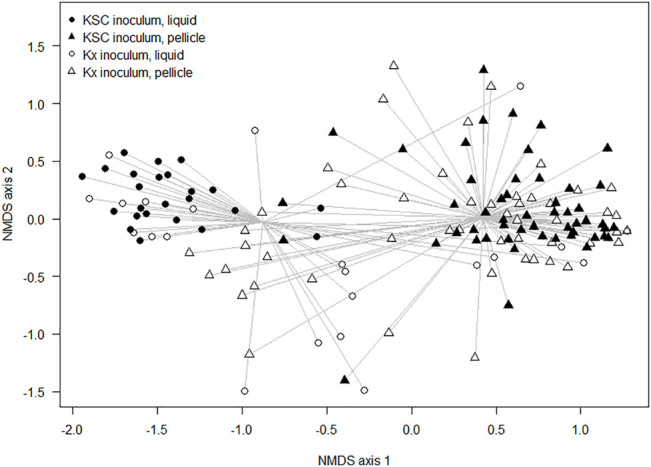
Non-metric multidimensional scaling ordination plot of composition differences of samples (inoculated with either the liquid or pellicle *K. xylinus*/Kombucha) using the Bray–Curtis dissimilarity index.

When considering the non-dominant composition data, there were significant differences between communities due to the inoculation type (ANOSIM: R = 0.28 and *p* < 0.001), during generations 0–2, and significant differences between cultures seeded from Kombucha or *K. xylinus* (ANOSIM: R = 0.14 and *p* < 0.001) across all generations. Figure 5 provides a visual illustration of these differences. The points represent the mean ordinations of the communities grown using liquid (circular points) or pellicle (triangular points) inocula, which were found to be significantly different (*p* < 0.001). Communities derived from a KSC inoculum (filled points) were found to be significantly different (*p* < 0.001) from those derived from a *K. xylinus* (KX) starter inoculum.

**FIGURE 6:**
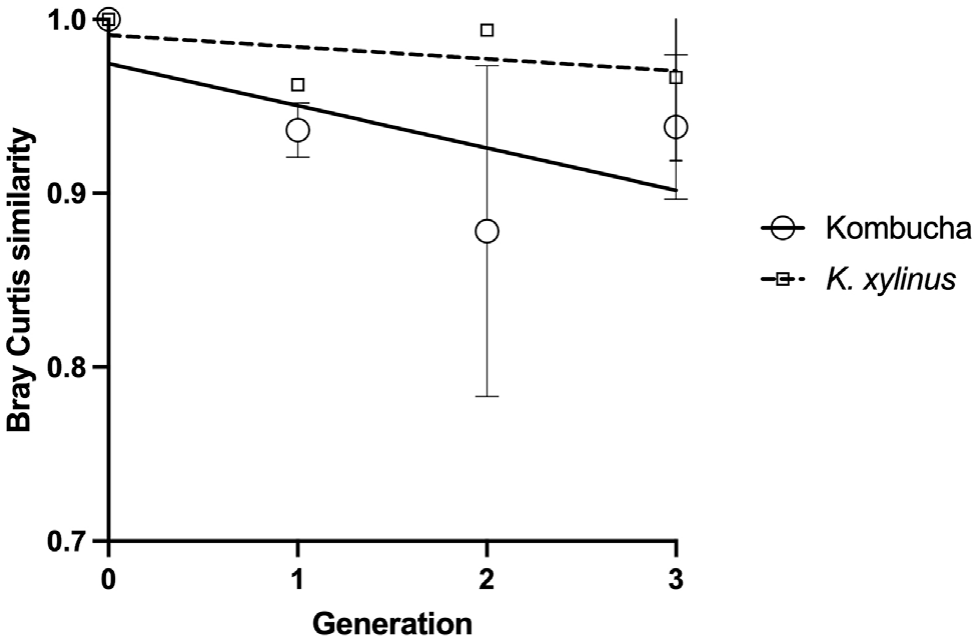
Trend of changes in the pellicle bacterial community relative to the original inoculant using the Bray–Curtis index.

**FIGURE 7:**
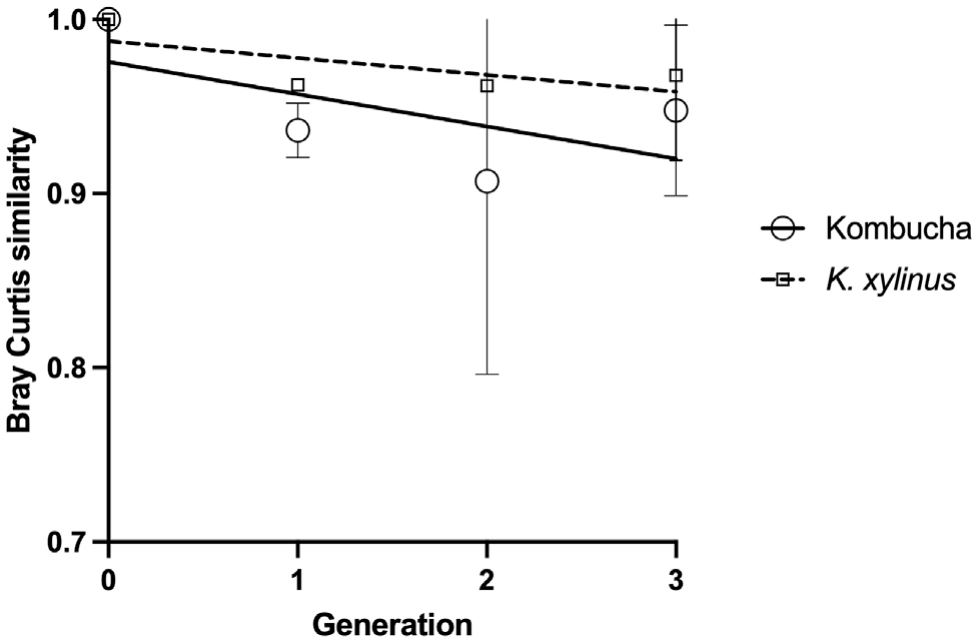
Trend of changes in pellicle bacterial community relative to previous generation using the Bray–Curtis index.

Using the Bray–Curtis similarity index to further explore and quantify the difference in the communities, a more marked drift away from the original pellicle community is noted in pellicles developed from a Kombucha inoculant (Figure 6) than those inoculated with *K. xylinus.* However, the rate of pellicle community composition change when compared to the previous generation is similar in trend for both Kombucha and *K. xylinus* (Figure 7).

Previous work studying the microbial communities in Kombucha tea liquid observed microbial community stabilization over time. In studies sampling tea broth, it was suggested that stabilization occurs after approximately 21 days (Chakravorty et al., 2016). This implies that stabilization of the community is an important factor when studying the BC pellicle-forming effects of both Kombucha and single isolates. Previous studies investigating the properties of the liquid culture consortium have suggested that there are critical times in fermentation at which the microbial community is most stable (Marsh et al., 2014; Chakravorty et al., 2016). However, it is important to note that some previous studies have used non-sterile sweetened black tea liquid as it is suggested the autoclave process can cause a build-up of toxins which inhibit the fermentation of Kombucha pellicles (Marsh et al., 2014). Further work will be required to tailor the methodology to ensure reproducibility of all aspects of BC production, for example, thickness of BC, and any specific properties required for commercialization. Therefore, it could be argued that the microbial diversity found in these studies was not adequately controlled. Nevertheless, the results of our study lead to the hypothesis that a community stabilization period’ (in the region of 30 days) is required to observe consistent, reproducible BC pellicle production.

## Conclusion

This study examined the changes of bacterial communities over several generations of sub-culturing (using either a Kombucha consortium or *Komagataeibacter xylinus* single isolate as a starter inoculum) to establish the reproducibility of the BC pellicle as a potential alternative textile for industrial use. *Komagataeibacter* (the genus responsible for the most prolific production of BC) was found to be the most abundant species in all samples tested. *Komagataeibacter xylinus* starter culture BC pellicle yield improved over subsequent generations; however, Kombucha starter cultures produced the highest yield of BC pellicles from generation 1. This leads to the hypothesis that it is the microbial and fungal community and extracellular polymeric substances in the Kombucha consortium that support more vigorous BC production, giving more stability to the BC-forming bacterial strains. It is also suggested that an increase of diversity negatively impacts the ability of the *Komagataeibacter* genus to produce BC in the case of single isolate inocula.

Additionally, this study has shown that it is a mix of pellicle and liquid inocula that gives the best yield of BC. As the microbial community is more mobile in a liquid than a solid pellicle, it is proposed that the BC-forming microbes can more easily access carbon sources and thus more quickly produce BC.

Reproducibility of the BC pellicle across generations is essential for effective applications across the fashion and biotechnology industries. We conclude that Kombucha starter cultures produce BC pellicles which are more reproducible across generations than those created from pure isolates of *K. xylinus*. However, the Kombucha community needs to reach a critical point to maximize the yield of BC production. The study suggests this may be after a minimum of two generations, but this could be confirmed by examination of further generations of sub-culturing.

The findings of this study suggest that BC pellicles produced from Kombucha starter cultures could provide a reproducible sustainable model for generating textile materials. Further works should now examine the effects of sub-culturing on performance properties of the BC pellicles to establish potential end uses for the material.

## Data Availability

Sequence data that support the findings of this study have been deposited in NCBI BioProject database with accession number SAMN23827663 to SAMN23827813.
